# Letter from a Deputy Editor

**DOI:** 10.1093/ehjdh/ztac034

**Published:** 2022-06-17

**Authors:** Peter de Jaegere

**Affiliations:** Thoraxcenter, Erasmusm University Rotterdam, Dr Molewaterplein 40, 3015 GD, Rotterdam, The Netherlands

This issue starts with a Letter to the Editor from Prof. Kohler (Free University Berlin) and an interview by Dr. R. van der Boon with Prof. B. Meder who holds professorship of ‘Precision Digital Health’ at the University of Heidelberg (Cardiopulse Digital). At first glance, one may wonder the alikeness, yet a gentle pause learns that both papers hit the very heart of our journal, thereby substantiating the clinical, scientific, and societal need and hopefully value of our beloved journal. Digital health (DH) reflects a vision and ambition that sails on the waves of incessant technological innovations in the field of data collection, transfer, stockage, and analysis. The vision and ambition are to foster and/or to improve health and well-being via subject-centred prevention and/or treatment of disease and disabilities that—from a broader perspective—must be affordable and accessible to all. As such, it demands a free and plural world in which individual and societal sovereignty are fundamental, which must unconditionally be respected and if not, strongly condemned and overpowered.

Accomplishment of the above is not easy. It is complicated by conditions that pointlessly take away human and financial resources needed to build a better world to the benefit of all, more so than a lack of technological ingenuity and solutions. With respect to medicine and all other domains of value to quality of life and survival of flora and fauna that includes humankind, we are dealing with huge volumes of multidimensional data of different physical nature that are collected by on-site or remote tools, their transfer, storage, analysis, and finally proper interpretation and reaction. The first two papers of this issue briefly touch upon this and are a warming up of the subsequent papers in this issue.

The implementation of DH (from data collection to analysis and decision-making) to effective and affordably reduce the burden of disease and/or disabilities is not an easy undertaking. A proper understanding of the intricacies of all constituents of the ‘DH-chain’ is mandatory. To start at the end of the chain, one must understand of how the findings of analyses came about for proper interpretation and ensuing reaction. This is in essence not different when using ‘regular statistics’ or interpreting the findings of randomised clinical trails. Yet, artificial intelligence (AI) for the assessment of associations between data is a bit more esoteric.

The paper of Al-Zaiti *et al*. is in this respect very welcome, and more of those are wanted since DH is complex spanning a vast array of domains that demands quite some Olympic brain gymnastics. As such the paper of Al-Zaiti *et al.* is tutorial and gaps the bridge between the preclinical (engineers, data, and computer scientists,) and clinical field. The checklist for the evaluation of the rigour and reproducibility of the four machine learning building blocks—data curation, feature engineering, model development, and clinical deployment—is informative and stimulates further contemplation.

This is followed by a series of 15 original papers that are exemplar of what has been discussed above. Two papers (Makimoto *et al.*, Lachmann *et al.*) are related to valvular heart disease and another six to electrophysiology. Van Dam *et al.* report on the application of CineECG to the 12-lead surface electrocardiogram (ECG) for the 3D visualization of atrial electrical activity during the PQ-interval, relating atrial electrical activity to the atrial anatomy. Five papers focus on the detection/prediction of atrial fibrillation (Afib) via AI-enabled analysis of the ambulatory ECG by Holter monitoring (*Singh et al.*) and clinical relevance of early detection of Afib (van Husen *et al.*, Gordon *et al.*, Stegmann *et al.*, Christopoulos *et al.*).

The role of AI in finding relationships between features stemming from electromagnetic waves (*ECG, MRI*) and patient-specific related features such as gender (van Es *et al.*) and clinical conditions such as hyperthyroidism (*Kwon et al.*) and pulmonary hypertension (Alabed *et al.*) is presented next. The series of original papers end with two on remote monitoring that may be used for continued care and treatment of the patient in his/her own environment or rehabilitation (Chun-Ka *et al.,* Miyazaki *et al.*), emphasizing the significance of telemedicine.

This issue ends with a short report (Konig *et al.*) and two review papers (van Spall *et al.*, Islam *et al.*), which focus on specific domains of DH and AI-enabled analysis in specific patient categories and purposes. Notwithstanding their focus, they indirectly deliver a wider awareness of what may be expected of DH in medicine.

Steven Weinberg (Noble price winner physics, 1979) once said ‘there is no limit to the human brain’. He may be right. We can solve whatever problem provided humankind has the will to do so. Yet, one may question who will benefit from DH and technological innovations lessening the burden of disease and/or disabilities given the increasing inequality in the fundamental needs of decent life between populations and regions in the world, so nicely discussed in ‘Doughnut Economics’ by Kate Raworth. So, on a more global scale, there is a lot at stake and, henceforth, to fulfill our dream of a better life and world. As Henry Kissinger said, ‘history of mankind is a succession of war and peace’. This will unfortunately continue as long as we—or some—do not or do not want to learn from history. At the dawn of the 6^th^ century BC, the Athens ‘invented’ a democratic constitution with (at that time) all free-men a say in government when tension between factions rose. At roughly the same time the Chinese embraced Kongfuzi, better known as Confucius, when authorities started battling for dominance. Quousque tandem abutere, Catilina, patientia nostra (Cicero)? Cicero will most likely address his discourse today to other people than Catilina.

We may hope that all and good mother earth, which is the cradle and preserve of life, may benefit from the fruits of science(s) that best thrives in the realm of freedom and respect for the pillars of life and society via debate, thesis, and antithesis. Via this journal, we seek to contribute the best we can. Gratitude goes to those who make this happen, especially the authors and reviewers. To give an idea of what it takes, the 15 original papers and two reviews of this issue have been reviewed by an average of three reviewers/paper (min 2, max 6) during an average of three rounds (min 1, max 6). We hope for a prosperous journey.

